# Effects of exercise types on white matter microstructure in late midlife adults: Preliminary results from a diffusion tensor imaging study

**DOI:** 10.3389/fnagi.2022.943992

**Published:** 2022-11-18

**Authors:** Feng-Tzu Chen, Hideaki Soya, Michael A. Yassa, Ruei-Hong Li, Chien-Heng Chu, Ai-Guo Chen, Chiao-Ling Hung, Yu-Kai Chang

**Affiliations:** ^1^Department of Sports Medicine, China Medical University, Taichung, Taiwan; ^2^Laboratory of Exercise Biochemistry and Neuroendocrinology, Faculty of Health and Sport Sciences, University of Tsukuba, Tsukuba, Japan; ^3^Sports Neuroscience Division, Department of Mind, Advanced Research Initiative for Human High Performance (ARIHHP), Faculty of Health and Sport Sciences, University of Tsukuba, Tsukuba, Japan; ^4^Department of Neurobiology and Behavior, University of California Irvine, Irvine, CA, United States; ^5^Center for the Neurobiology of Learning and Memory, University of California Irvine, Irvine, CA, United States; ^6^Department of Physical Education and Sport Sciences, National Taiwan Normal University, Taipei, Taiwan; ^7^College of Physical Education, Yangzhou University, Yangzhou, China; ^8^Masters in Sport Facility Management and Health Promotion, National Taiwan University, Taipei, Taiwan; ^9^Department of Athletics, National Taiwan University, Taipei, Taiwan; ^10^Institute for Research Excellence in Learning Science, National Taiwan Normal University, Taipei, Taiwan

**Keywords:** cognitive function, exercise type, fractional anisotropy, white microstructure, Tai Chi, Taijiquan

## Abstract

Higher aerobic fitness during late midlife is associated with higher white matter (WM) microstructure. Compared with individuals engaged in irregular exercise, those who engage in regular aerobic exercise show higher fractional anisotropy (FA), a diffusion tenor imaging (DTI) measure that provides an index of WM microstructural integrity. However, whether other types of exercise, such as Tai Chi, can also facilitate WM changes in adults during late midlife remains unknown. The present study compares two types of exercise, Tai Chi and walking, with a sedentary control group, in order to examine the effects of exercise on WM microstructure and determine the regional specificity of WM differences. Thirty-six healthy adults between the ages of 55 and 65 years participated in the study. Based on the participants’ exercise habits, they were allocated into three groups: Tai Chi, walking, or sedentary control. All participants were required to complete physical fitness measurements and completed magnetic reasoning imaging (MRI) scans. Our results revealed that the Tai Chi group exhibited a higher FA value in the left cerebral peduncle, compared to the sedentary control group. We also observed that both the Tai Chi and walking groups exhibited higher FA values in the right uncinate fasciculus and the left external capsule, in comparison to the sedentary control group. Increased FA values in these regions was positively correlated with higher levels of physical fitness measurements (i.e., peak oxygen uptake [VO_2_peak], muscular endurance/number of push-up, agility, power). These findings collectively suggest that regular exercise is associated with improved WM microstructural integrity, regardless of the exercise type, which could guide the development and application of future prevention and intervention strategies designed to address age-related cognitive impairments during late midlife.

## Introduction

White matter (WM) microstructure begins to show evidence of significant decline as early as late midlife, which may lead to disruptions in neural communication and increase the risk of developing age-related cognitive decline or neurodegenerative diseases, such as Alzheimer’s disease (AD; Damoiseaux et al., 2009; Madden et al., 2009; Zarei et al., 2009; Filley and Fields, 2016). Fractional anisotropy (FA), derived from diffusion tensor imaging (DTI), represents a general index of WM microstructural integrity (Raz et al., 2005), and decreased FA values are associated with global brain atrophy, hypometabolism, and diminished activation and are often accompanied by impaired cognitive function (Charlton et al., 2006; Kennedy and Raz, 2009; Lebel et al., 2012; Sexton et al., 2014). Taken together, the significance of maintaining WM microstructure to support cognitive function has highlighted the importance of preventing or alleviating age-related declines in microstructural integrity among adults in the late midlife stage.

Growing evidence suggests that a high level of physical activity (PA) represents a promising candidate as an intervention for reducing or delaying age-related WM microstructure deterioration (Gons et al., 2013). Cross-sectional studies have indicated that aerobic fitness, in particular, enhanced through the performance of various types of aerobic exercise, including walking or running, is significantly associated with increased FA values, compared with those for inactive individuals or those with poor aerobic fitness (Tseng et al., 2013; Oberlin et al., 2016). Randomized control trials indicated that FA values were significantly altered after aerobic exercise training (Voss et al., 2013; Sexton et al., 2020), and observed that WM benefits were considered evidence sufficient to support an intact central nervous system and cognitive improvements in studies examining the effects of aerobic exercise training among older adults (Colcombe et al., 2004, 2006; Mendez Colmenares et al., 2021). Furthermore, a systematic review including 29 studies supports this viewpoint, indicating a positive relationship between aerobic exercise and WM integrity (Sexton et al., 2016). These findings collectively show that aerobic exercise exerts potentially beneficial effects on WM maintenance. However, the benefits seemed incapable of affecting other indices of brain volume (e.g., grey volume, cortical thickness) (Pani et al., 2021), or improving special populations (e.g., people with AD; Venkatraman et al., 2020).

Despite the wealth of data indicating a positive relationship between aerobic exercise and WM integrity, substantially less information exists regarding the benefits associated with other types of exercise, such as Tai Chi, also known as Tai Chi Chuan or Taijiquan, a multimodal mind–body exercise that integrates mindfulness with PA, mental stimulation, and deep breathing and can provide social interaction when practiced in a group setting (Chang et al., 2010; Wang et al., 2010, 2018). Tai Chi has been shown to effectively enhance multiple indicators of physical fitness, including muscular endurance, agility, and power (Zou et al., 2017, 2019), and Tai Chi has been suggested to potentially enhance cognitive performance and facilitate brain activation (Tao et al., 2016; Wu et al., 2018; Liu et al., 2019; Yue et al., 2020). The beneficial effects of Tai Chi on brain structure and function were reported in a recent systematic review (Chen et al., 2020b); however, the effects of Tai Chi on WM microstructure remain poorly understood.

Previous studies examining the effects of Tai Chi have not reported differences in WM microstructure (Pan et al., 2018), and whether Tai Chi exerts regional specificity with regard to WM microstructure also remains unknown. To fully understand how WM microstructure differences in response to the practice of Tai Chi, we can extrapolate from other PA studies that highlight differences in WM microstructures of specific brain regions. For example, a recent study focused on late middle-aged adults (mean age = 54 years) demonstrated that aerobically trained participants exhibited higher FA values in the anterior and superior cortex than those with sedentary lifestyles (Tarumi et al., 2021). Although evidence from late middle-aged adults remains, studies conducted in older adults (≥65 years) have also emphasized the beneficial effects of PA on WM microstructure of specific brain regions, including the corpus callosum (Yao et al., 2019), the corona radiata (Ruotsalainen et al., 2020; Strömmer et al., 2020), the hippocampal cingulum (Harasym et al., 2020), and the uncinate fasciculus (Tseng et al., 2013; Krafft et al., 2014; Schaeffer et al., 2014).

In the present study, we addressed these important knowledge gaps regarding the role played by WM microstructural integrity in the relationship between PA and brain health. No studies have investigated the effects of different types of exercise on WM microstructure using DTI parameters or identified regions susceptible to beneficial differences in response to the performance of Tai Chi or aerobic exercise during late midlife. We compared WM microstructure parameters in groups of adults who practiced either Tai Chi or walking with those observed in sedentary controls and determined the regional specificity of WM microstructural differences between these groups. We hypothesized that both Tai Chi and walking would be associated with increased FA values compared with irregular exercise, and we expected to observe significant differences in WM measurements obtained in the frontal and temporal regions (Colcombe et al., 2006). In supplementary analyses, the study also investigated whether regions with significant differences in FA values were associated with different physical fitness parameters, such as aerobic fitness, muscular strength, muscular endurance, flexibility, body fat percentage, agility, or power. Based on prior studies (Tseng et al., 2013; Oberlin et al., 2016; Yao et al., 2019), we predicted that a correlation would exist between regions showing differences in FA values and specific physical fitness parameters associated with the performance of either Tai Chi (muscular endurance, agility, and power) or walking (aerobic fitness).

## Materials and methods

### Participants

A total of 36 healthy adults between the ages of 55–65 years (mean [M] = 57.89 years; standard deviation [SD] = 3.52 years) were recruited via flyers posted in communities. Hospitals, universities, and sport clubs. All recruited participants were required to meet additional criteria based upon a self-reported exercise experience questionnaire. Specifically, Participants in a Tai Chi (*n* = 12) or a walking group (*n* = 12) participated their respective exercises at least three times per week for 60 min per session, with moderate to vigorous intensity for the previous 3 months, and participants in a sedentary control group (*n* = 12) were those who participated less than two times per week for 60 min per session, with moderate to vigorous intensity, for previous 3 months. Eligible participants were excluded if they presented any evidence from doctors of existing cognitive dysfunction (e.g., AD, any type of dementia) or physical disease (e.g., untreated hypertension or chronic heart disease). The current study was approved by the Institutional Review Board at the National Taiwan University, and all participants completed written informed consent prior to enrollment.

### Physical fitness measurements

Health-related and skill-related fitness were examined as indicators of physical fitness. Health-related fitness consisted of aerobic fitness, muscular strength, muscular endurance, flexibility, and body fat percentage, whereas skill-related fitness included agility and power.

Aerobic fitness was assessed on an ergometer (Ergoselect 100/200; Ergoline GmbH, Germany), using a maximal graded exercise test, according to the Young Men’s Christian Association (YMCA) protocol (Golding, 2000). The YMCA protocol includes three consecutive stages, each consisting of 3 min of cycling. During the initial stage, participants cycled at a power of 25 W using a pedaling rate of 50 rpm for 3 min. Heart rate (HR) was continuously monitored, and HR recordings for the last 15–20s of each stage were used to determine the workload for the next two stages. The increased workloads performed during the second and third stages were based on the adjusted HR measured during the previous stage. For example, if a participant’s HR during the initial stage was less than 80 bpm, the power increased to 125 W (750 kpm/min) during the second stage, followed by 150 W (900 kpm/min) during the third stage. Peak oxygen uptake (VO_2peak_) was calculated by combining the participant’s body weight and age-predicted maximal HR (calculated as 220 − age) (American College of Sports Medicine, 2018).

Muscular strength for each hand was determined using a handgrip dynamometer, and muscular endurance was assessed using two approaches: 60-s push-ups for men or 60-s bent-knee push-ups for women, and 60-s abdominal sit-ups. Flexibility was assessed using the sit-and-reach test to test the ductility of the lower back and hamstring. Body fat percentage was calculated via a bioimpedance spectroscopy instrument (InBody 3.0 DS12B887, Dallas, TX, United States). Agility was assessed using the *T* test, which requires participants to jump around cones arranged in a *T* shape as quickly as possible and measures the time required to complete the task, with a lower time indicating better performance. Power was determined via a vertical jump test, in which participants were asked to jump as high as possible within a circular area.

### DTI acquisition and analysis

MRI scans were obtained using a 3 Tesla Siemens TIM TRIO scanner (Erlangen, Germany). All images were obtained via a single-shot spin-echo echo planar imaging sequence with following parameters: Repetition time (TR) = 11,000 ms; echo time (TE) = 104 ms; flip angle = 90°; field of view (FOV) = 230.4 × 230.4 mm; matrix size = 128 × 128; voxel size: 2 mm × 2 mm × 2 mm; 70 axial oblique slices with 2-mm gaps; 30 noncollinear directions of *b* = 0 and 1,000 s/mm^2^; non–diffusion-weighted image of b = 0 mm^2^. All images were examined by technical operators and we excluded any images with incidental noise.

All DTI images were preprocessed via Tract-Based Spatial Statistics (TBSS; Smith et al., 2006), which included (a) motion and eddy correction of the images and corresponding b-vectors, (b) removal of the skull and non-brain tissue using the Brain Extraction Tool (Smith, 2002), and (c) computation of voxel wise eigenvalues and eigenvectors of diffusion parameters via local fitting of the diffusion tensor model. Then, the nonlinear registered FA images (FMRIB58_FA; Rueckert et al., 1999) were averaged to compute a mean FA image and an average skeleton with threshold at an FA of 0.2 was generated in order to represent a center of white matter trajectories (Smith et al., 2004, 2006). Afterward, the normalized FA image was projected onto a mean FA skeleton and these FA images were evaluated for FA values.

**Figure 1:**
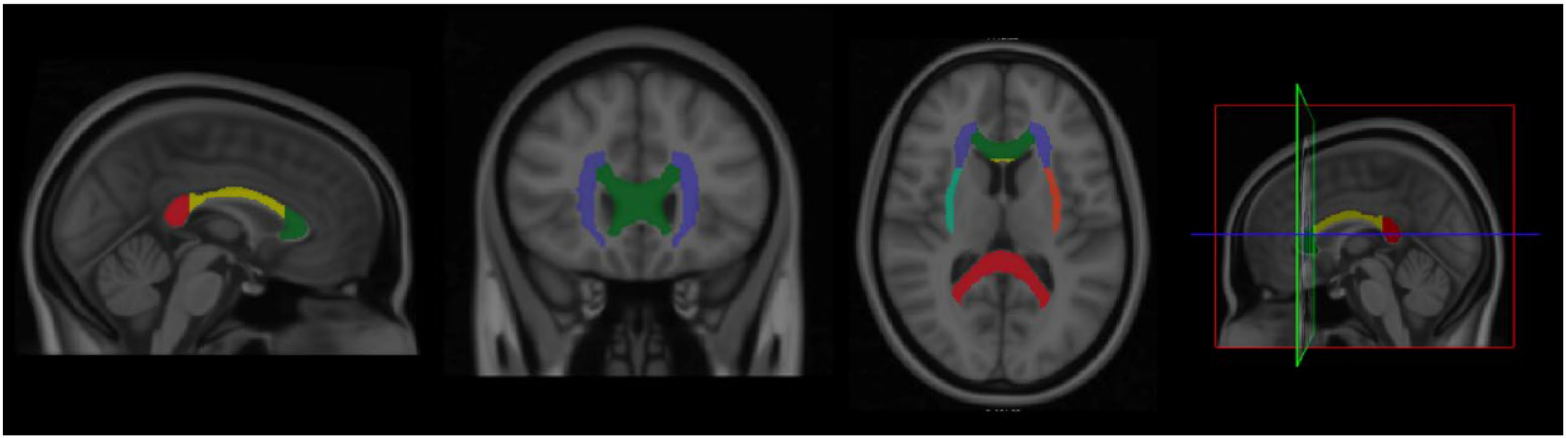
The selection of regions of interests (ROIs).

The whole tracts selection was followed from the JHU ICBM-DTI-81 white matter atlases and we theoretically selected seven tracts based on previous findings (Tseng et al., 2013; Krafft et al., 2014; Schaeffer et al., 2014; Xiong et al., 2018; Yao et al., 2019; Harasym et al., 2020; Ruotsalainen et al., 2020; Strömmer et al., 2020; Tarumi et al., 2021), including (1) corpus callosum, (2) bilateral cerebral peduncle, (3) bilateral anterior corona radiata, (4) bilateral superior corona radiata, (5) the bilateral posterior corona radiata, (6) the bilateral external capsule, and (7) the bilateral uncinate fasciculus (A total of 7 references ROI were selected; Figure 1).

### Procedure

All eligible participants were required to visit our laboratory two times. During the first visit, the International Physical Activity Questionnaire (IPAQ), an international surveillance questionnaire used to assess PA levels, was administered to characterize the participants’ overall lifestyles as sedentary, lightly active, moderately active, or vigorously active (Bauman et al., 2009). Then, the digit span task from the Wechsler Adult Intelligence Scale, Third Edition (*WAIS-III*) (Wechsler, 1997), including the digit forward and backward span, was used to evaluate the working memory component of intelligence (Chang et al., 2017; Wu et al., 2019). In the second visit, the magnetic reasoning imaging (MRI) scan was recorded, and the scanning time was approximately 1 h. Following the completion of an MRI scan, physical fitness was measured by trained examiners, including health-related fitness assessments (aerobic fitness, muscular strength, muscular endurance, flexibility, and body fat percentage) and skill-related fitness assessments (agility, power).

### Statistical analysis

Analysis of variance (ANOVA) was performed using SPSS software to analyze differences in participants demographics, exercise experience, and physical fitness measures among the three groups (Tai Chi, walking, and sedentary control). Additionally, one-way ANOVA was used to compare FA values among the three groups and significant differences were assessed by *post hoc* least significant difference (LSD) analysis for pairwise comparisons and set *p* values at 0.05.

After checking the normality via distribution test (>0.05), we used two-trailed Pearson correlation test without controlling covariates to examine the correlations between physical fitness and FA values. All *t* values of Pearson’s correlation were computed by the formula [t = r * √n-2/√1-r^2^] with considering degree of freedom (n−2) and *p* values less than 0.05 were considered significant.

## Results

### Participant demographics

Table 1 summarizes the participants’ characteristics for the three groups (Tai Chi, walking, and sedentary control). No significant differences were observed among groups for age, weight, education level, or income level (*ps* > 0.05). Additionally, no significant differences were observed for the digit span forward and digit span backward performance between groups (*ps* > 0.05).

**Table 1:** Description of participants characteristic, exercise experience, and health-and skill-related fitness among three groups.

Variable	Group			Total
	Tai Chi	Walking	Sedentary control	
**Participant characteristic**				
Sample/Female (%)	12/5 (42%)	12/7 (58%)	12/9 (75%)	36/21 (58%)
Age (year)	58.33 ± 4.23	57.42 ± 2.87	57.92 ± 3.58	57.89 ± 3.52
Weight (kg)	64.51 ± 9.51	61.76 ± 7.97	67.41 ± 8.47	64.56 ± 8.74
Education (year)	13.08 ± 3.20	13.17 ± 3.01	13.92 ± 3.48	13.39 ± 3.23
Income (US dollar)	972.22 ± 459.65	1000.00 ± 402.02	805.56 ± 388.17	925.93 ± 414.89
Digit span forward	12.67 ± 1.97	12.58 ± 2.31	11.42 ± 2.57	12.22 ± 2.31
Digit span backward	6.67 ± 2.06	7.00 ± 2.17	5.67 ± 1.83	6.44 ± 2.05
**Exercise experience**				
IPAQ (MET)	3390.00 ± 668.01^*^	3355.83 ± 2,553.90^*^	2417.50 ± 3,033.21	3054.44 ± 2300.12
Regular exercise (yrs)	4.00 ± 1.65^*^	3.00 ± 1.28^*^	0.08 ± 0.29	2.36 ± 2.06
**Health-related fitness**				
VO_2peak_ (mL/kg/min)	39.27 ± 5.82 ^*^	44.47 ± 9.64^*^	28.03 ± 5.87	37.25 ± 9.96
Muscular strength	65.08 ± 20.75 ^*^	72.03 ± 19.50^*^	50.75 ± 17.93	62.64 ± 20.91
Muscular endur/press up	11.58 ± 11.57 ^*^	18.42 ± 9.28^*^	2.08 ± 3.58	10.69 ± 10.93
Muscular endur/ASU 30	10.58 ± 7.08 ^*^	12.50 ± 7.49^*^	3.92 ± 5.04	9.00 ± 7.43
Muscular endur/ASU 60	18.50 ± 11.95^*^	22.33 ± 13.67^*^	5.33 ± 7.39	15.38 ± 13.24
Flexibility (cm)	42.29 ± 6.09 ^*^	35.67 ± 9.97^*^	26.42 ± 11.70	34.79 ± 11.38
Body fat mass	27.42 ± 5.54 ^*^	23.11 ± 4.29^*^	31.68 ± 6.22	27.40 ± 6.34
**Skill-related fitness**				
Agility (ms)	17.49 ± 3.42 ^*^	17.32 ± 1.93^*^	25.01 ± 5.78	19.94 ± 5.34
Power (cm)	28.50 ± 7.40^*^	36.67 ± 14.24^*^	21.25 ± 8.09	28.81 ± 11.93

IPAQ, international physical activity questionnaire; Muscular endur., muscular endurance; ASU 30, abdominal sit-ups for 30s; ASU 60, abdominal sit-ups for 60s; * represents a significant difference compared to the sedentary control group (*p* < 0.05).

### Exercise experience

Table 1 summarizes participants’ exercise experience among three groups. As confirmation of the correct characterization of exercise and sedentary control groups, a significant group difference for IPAQ scores was observed in two exercise group compared to sedentary control group, but no significant difference between Tai Chi and walking group (*p* > 0.05). Additionally, a significant group difference was observed in the number of that participants who reported participating in regular exercise [*F*(2,33) = 33.53, *p* < 0.01, partial η2 = 0.67]. Follow-up analysis revealed that the Tai Chi and walking groups reported more years engaged in regular exercise than the sedentary control group.

### Physical fitness

Table 1 summarizes the health-and skill-related fitness parameters across the three groups. When assessing health-related fitness variables, significant group differences were observed for VO_2peak_ [*F*(2,33) = 15.76, *p* = 0.00, partial η2 = 0.49], muscular strength [*F*(2,33) = 3.76, *p* = 0.03, partial η2 = 0.19], muscular endurance for push-ups [*F*(2,33) = 10.40, *p* = 0.00, partial η2 = 0.39], muscular endurance for sit-ups for 30 s [*F*(2,33) = 5.56, *p* = 0.01, partial η2 = 0.25], for sit-ups for 60s [*F*(2,33) = 7.45, *p* = 0.02, partial η2 = 0.31], flexibility [*F*(2,33) = 8.38, *p* = 0.00, partial η2 = 0.34], and fat mass percentage [*F*(2,33) = 7.53, *p* = 0.00, partial η2 = 0.31]. The follow-up analysis revealed that the Tai Chi and walking groups exhibited higher performance than the sedentary control group for all health-related fitness indexes, with no significant differences between the two exercise-performing groups (*ps* > 0.05). When assessing skill-related fitness variables, significant group differences were observed for agility [*F*(2,33) = 14.26, *p* = 0.00, partial η2 = 0.46] and power [*F*(2,33) = 6.63, *p* = 0.00, partial η2 = 0.29]. The follow-up analysis revealed that the Tai Chi and walking groups exhibited higher performance than the sedentary control group for all skill-related fitness indexes, with no significant difference between the two exercise-performing groups (*ps* > 0.05).

### FA values

**Figure 2:**
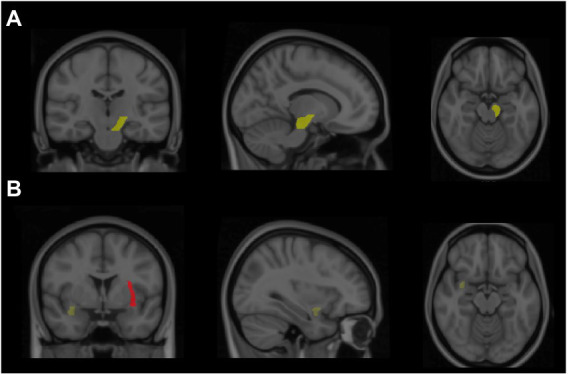
Highlight the greater fractional anisotropy (FA) value of brain regions from both Tai Chi and walking groups compared to the sedentary control group. **(A)** Revealed that the brain region of the Tai Chi group exhibited higher FA value in the left cerebral peduncle. **(B)** Showed in brain regions that the Tai Chi or walking groups displayed higher FA value in right uncinate fasciculus (yellow) and left external capsule (red), respectively.

The results indicated a significant group difference in the FA values of the left cerebral peduncle [*F*(2,33) = 3.17, *p* < 0.05, partial η2 = 0.16]. The follow-up analysis revealed that the Tai Chi group had a larger FA value than the sedentary control group (*p* < 0.05; Figure 2), with no significant difference observed between the Tai Chi and walking groups (*p* > 0.05). Additionally, the results revealed a significant group difference in the FA values of the left external capsule [*F*(2,33) = 3.78, *p* = 0.03, partial η2 = 0.19]. The follow-up analysis revealed that the Tai Chi and walking groups had larger FA values than the sedentary control group (*ps* < 0.05; Figure 2), and no significant difference was observed between the Tai Chi and walking groups. The results also revealed a significant group difference in the FA values of the right uncinate fasciculus [*F*(2,33) = 4.61, *p* = 0.02, partial η2 = 0.22]. The follow-up analysis revealed that the Tai Chi and walking groups had larger FA values than the sedentary control group (*ps* < 0.05; Figure 2), with no significant difference observed between the Tai Chi and walking groups.

No significant group differences were observed for the corpus callosum, right or left cerebral peduncle, right corona radiata (anterior, superior, or posterior), right external capsule, or left uncinate fasciculus.

### Correlations between physical fitness parameters and FA values

**Figure 3:**
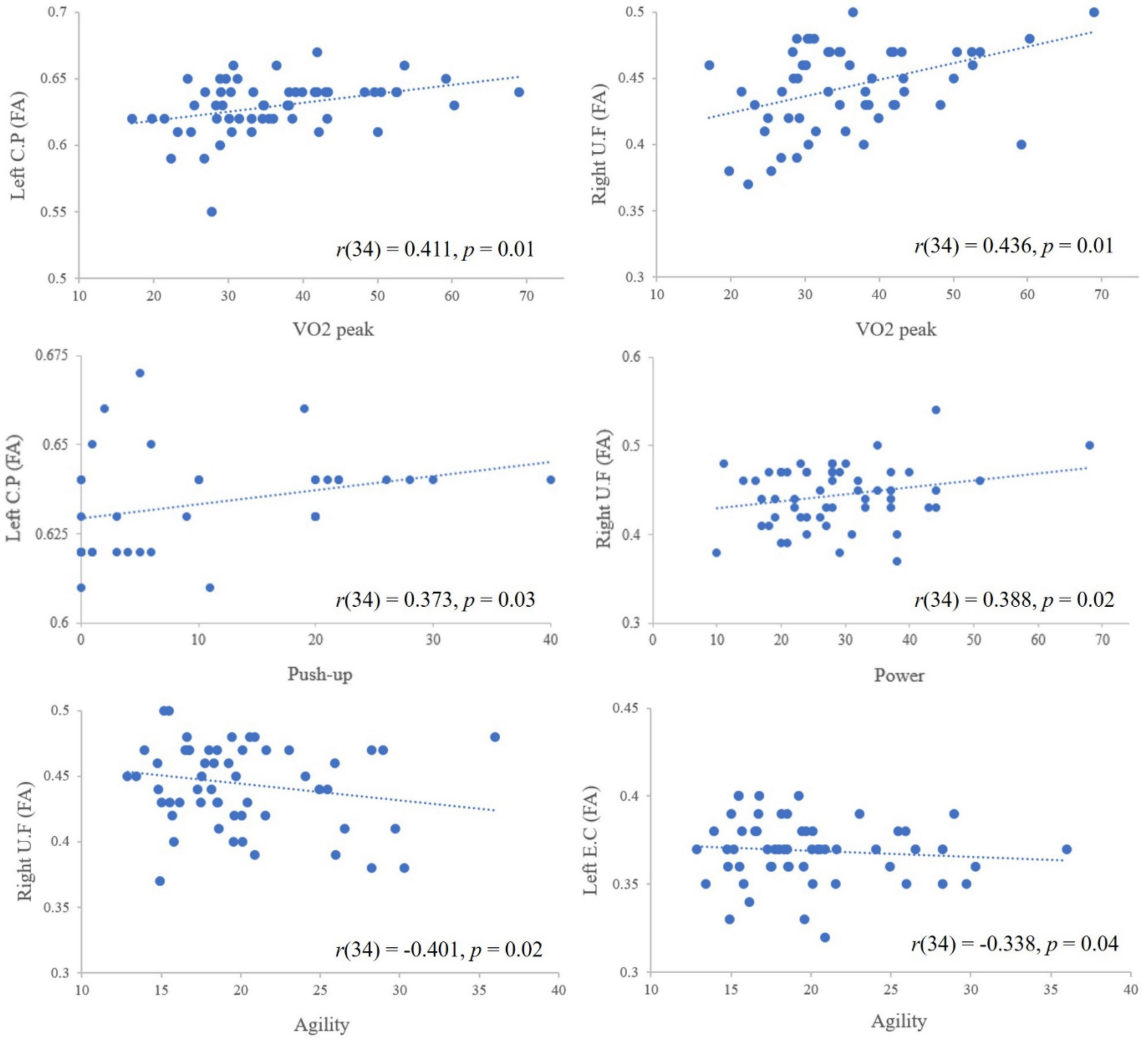
The correlations between physical fitness measurements and FA value. The dotted lines represent the trend lines (i.e., positive, negative trend) and the negative sloped trendlines were appeared between agility and FA values because agility was determined by *T* test (lesser time represents greater agility ability); CP, cerebral peduncle; UF, uncinate fasciculus; EC, external capsule.

Pearson’s correlation analysis revealed that a VO_2peak_ value was significantly correlated with high FA values in the left cerebral peduncle [*r*(34) = 0.411, *p* = 0.01] and the right uncinate fasciculus [*r*(34) = 0.436, *p* = 0.01]. The results also showed that a correlation between push-up performance and the FA value in the left cerebral peduncle [*r*(34) = 0.373, *p* = 0.03], between agility and the FA values in the right uncinate fasciculus [*r*(34) = −0.401, *p* = 0.02] and the left external capsule [*r*(34) = −0.338, *p* = 0.04], and between power and the FA value in the right uncinate fasciculus [*r*(34) = 0.388, *p* = 0.02; Figure 3).

## Discussion

This study investigated whether different exercise types are associated with WM microstructure changes in late middle-aged adults. We therefore examined FA value as WM integrity in various brain regions of those who engaged in regular Tai Chi practice (Tai Chi group), regular aerobic exercise (walking group), or irregular exercise (sedentary control group). Our findings revealed that the Tai Chi group exhibited a significantly higher FA value in the left cerebral peduncle than the sedentary control group. We also observed that both the Tai Chi and walking groups exhibited higher FA values in the right uncinate fasciculus and the left external capsule than the sedentary control group, implying that engaging in these exercise types is associated with higher WM in some regions of both the right and left hemispheres. Furthermore, we observed that better performance on various physical fitness parameters was associated with higher FA values, including a positive correlation between VO_2peak_ and the FA values in the left cerebral peduncle and the right uncinate fasciculus, between push-up performance and the FA value in the left cerebral peduncle, between agility and the FA values in the right uncinate fasciculus, and between power and the FA value in the right uncinate fasciculus.

The present findings provide evidence suggesting that individuals engaged in regular Tai Chi exercise exhibited a higher FA value in the left cerebral peduncle than the sedentary control group, but no significant difference on FA between walking and sedentary control group in the brain region. In general, higher FA values are associated with an increase in tissue anisotropy (Sen and Basser, 2005), which serves as an indicator of stabilized myelin integrity (Burzynska et al., 2010). The present findings suggest that Tai Chi and walking may have different effects on the neural mechanisms associated with the cerebral peduncle, as indicated by the differences in FA values, and that Tai Chi group may result in more beneficial effects on FA values than walking group in this brain region. The cerebral peduncle is a crucial region for the performance of life skills involving various motor and sensory functions, such as motor control and coordination (Jane et al., 1968). Previous studies have shown that individuals who engaged in exercises that combine cognitive and motor training (i.e., basketball training) demonstrated higher FA value in the cerebral peduncle than control individuals on a wait-list (Cai et al., 2020). Although previous studies have demonstrated the beneficial effects of Tai Chi on brain functions, such as improved cerebral blood flow and enhanced functional connectivity (Tao et al., 2016; Liu et al., 2019; Yue et al., 2020), to the best of our knowledge, the present study is the first to extend this prior knowledge to indicate that Tai Chi performed by late middle-aged adults may preserve the WM microstructure of the cerebral peduncles.

Individuals who engaged in both exercise types, Tai Chi or walking, in our study displayed higher FA values in the right uncinate fasciculus than the sedentary control group, a region known for its role in episodic memory, language formation, and mnemonic associations (Sato et al., 2012; Von Der Heide et al., 2013). A previous study suggested that significant differences can be observed in WM microstructure after a 6-month dance intervention, combining physical, cognitive, and social engagement, especially significant differences in the FA values in the anterior cortex (Burzynska et al., 2017), where the uncinate fasciculus is located. Similar to the exercise features of dance, Tai Chi is considered a complex PA that involves both physical fitness and cognitive engagement, and the combination of PA involving both motor and cognitive components has been shown to exert beneficial effects on WM microstructure. Another study focused on 6-month aerobic exercise training in a large sample (*n* = 264) of subjects between the ages of 57 and 86 years, which also suggested that exercise intervention resulted in significant change in the FA value in the uncinate fasciculus (Clark et al., 2019). The current findings in a population of late middle-aged adults (55 to 65 years) provide additional support that aerobic exercise (walking) has benefits on WM microstructure of the uncinate fasciculus. This finding is in accordance with a randomized control design with a 6-month aerobic exercise intervention, showing that an increase in the uncinate fasciculus was observed (Predovan et al., 2021). Taken together, the uncinate fasciculus appears to benefit from exercise engagement, regardless of the type of exercise performed, and both walking and Tai Chi may represent effective treatments for the prevention of age-related declines in the frontotemporal region.

The present study also observed higher FA values for both the Tai Chi and walking group than for the sedentary control group in the left external capsule. The external capsule, located between the putamen medially and the claustrum laterally, conveys fibers from sensorimotor, cognitive, and limbic regions of the cerebral cortex to the areas of the striatum, and previous neuroimaging studies have shown that the external capsule region is associated with language (e.g., verbal repetition and verbal fluency) (Dick et al., 2014) and cognitive functions (e.g., attention and executive function) (Schmahmann et al., 2008). Although few studies to date have reported evidence to suggest that regular exercise increases FA values in the left external capsule, the present study adds to the literature by providing the first evidence supporting that the performance of regular Tai Chi and walking exercise can facilitate the improvement of WM microstructure in the left external capsule.

Some regions in our study exhibited no significant differences in FA for the exercise groups relative to the sedentary control group, including the corpus callosum and corona radiata. Exercise may be less able to influence WM microstructure in the corpus callosum and corona radiata among adults in late middle-aged adults (i.e., 50–65 years old) because these regions may be relatively stable for this age group, whereas older adults (i.e., over 65 years old) are more sensitive to the beneficial effects of exercise on WM in these regions (Yao et al., 2019; Ruotsalainen et al., 2020; Strömmer et al., 2020). Therefore, age may be a factor that influences the magnitude of WM declines during the aging process, and focusing on multiple age ranges is recommended in future studies.

Consistent with our hypothesis, we observed that higher levels of aerobic fitness (VO_2peak_) were associated with higher FA values in the left cerebral peduncle and the right uncinate fasciculus. These findings demonstrate the beneficial effects for WM integrity associated with higher levels of aerobic fitness. Consistently, in our cross-sectional sample of late middle-aged adults (55–65 years), a positive association between aerobic fitness (VO_2peack_) and the FA value in the left cerebral peduncle was previously reported (Chen et al., 2020a). Previous studies have reported that several brain regions are sensitive to aerobic fitness, including the cingulum bundle, the corpus callosum, the anterior internal capsule, the fornix, and the anterior corona radiata (Johnson et al., 2012; Tseng et al., 2013; Hayes et al., 2015; Oberlin et al., 2016), and the present findings expand these associations to other regions, especially the cerebral peduncle and the uncinate fasciculus. In addition to aerobic exercise, other measures of physical fitness (i.e., push-up performance, agility, and power) were also positively correlated with FA values in specific regions in the present study, including correlations between push-up performance and the FA value in the left cerebral peduncle, between agility and the FA values in the right uncinate fasciculus and the left external capsule, and between power and the FA value in the right uncinate fasciculus. These measurements of physical fitness appear to be improved by both types (Tai Chi and walking), and the performance of regular physical fitness by adults in midlife may prevent the age-related deterioration of WM fibers and decrease the risks of neurogenerative disease development later in life.

Future studies should consider several limitations associated with the present study to better investigate the relationship between exercise types and DTI measures of WM microstructure in late midlife. First, we acknowledge that biological sex could influence the generalizability of the finding; however, the sex ratios among the three groups of the present study were uneven, and we were therefore unable to determine how sex affected the outcome measures. Furthermore, it should be noted that women included in study may be post-menopausal, which might be a factor to affect WM microstructure. Therefore, biological sex should be considered for the following studies. Second, the cross-sectional nature of our study limits conclusions regarding the causal associations between exercise and the maintenance of WM microstructure, and future randomized longitudinal studies remain necessary in order to understand the relationships between exercise types and WM microstructure in healthy adults during late midlife. Third, the present study was limited to DTI measure and only focused on FA value, rather than other areas [i.e., axial diffusivity (AD), radial diffusivity (RD), mean diffusivity (MD)], which might have reduced the generalizability of the finding (Mori and Zhang, 2006). Therefore, we suggest future studies should integrate multiple measures (e.g., WM and grey matter) or multiple DTI indices. Finally, a relatedly small sample size seems to limit the statistical power, and multiple comparisons increase the chance of a type 2 error, so we suggest the use of a multiple comparison correction with a large sample size for follow-up studies. A strength of the present study includes the use of physical fitness measures to ensure the characteristics of the Tai Chi and walking groups, and further compares these two exercises effects on WM with the sedentary control group, for investigating its difference on WM integrity influence.

## Conclusion

The present study was to investigate the relationships between exercise types and the preservation of WM microstructural integrity in late midlife, suggesting that late middle-aged adults who participate in regular Tai Chi demonstrate higher WM microstructural integrity in the left cerebral peduncle than those who do not engage in regular exercise. Additionally, engaging in both Tai Chi and aerobic exercise may activate neural mechanisms associated with improved WM microstructural integrity in the right uncinate fasciculus and the left external capsule. We also found that measurements of physical fitness associated with both Tai Chi and aerobic exercise were correlated with improved WM microstructural integrity. These findings could guide the development of future prevention or intervention strategies for combating age-related impairments in adults during late midlife.

## Data availability statement

The original contributions presented in the study are included in the article/supplementary material, further inquiries can be directed to the corresponding authors.

## Ethics statement

The studies involving human participants were reviewed and approved by Institutional Review Board at National Taiwan University. The patients/participants provided their written informed consent to participate in this study.

## Author contributions

F-TC, HS, MY, A-GC, C-HC, and Y-KC contributed to the conception of the work. F-TC, C-LH, C-HC, and Y-KC contributed to the design of the work. F-TC, R-HL, and C-LH conducted the literature search and statistical analysis. F-TC, HS, MY, and Y-KC wrote the first draft of the manuscript with support from R-HL, C-LH, A-GC, and C-HC. All authors contributed to the manuscript revisions and agreed with final approval of the version.

## Funding

This research work was supported by part of a grant from China Medical University (CMU110-N-26) and National Science and Technology Council (MOST 111-2410 H-039-012) in Taiwan to F-TC, and National Science and Technology Council in Taiwan (MOST 107-2628-H-003-003-MY3; MOST 110-2410-H-003-142 -MY3), and National Taiwan Normal University from the Higher 500 Education Sprout Project by the Ministry of Education (MOE) in Taiwan to Y-KC.

## Conflict of interest

The authors declare that the research was conducted in the absence of any commercial or financial relationships that could be construed as a potential conflict of interest.

## Publisher’s note

All claims expressed in this article are solely those of the authors and do not necessarily represent those of their affiliated organizations, or those of the publisher, the editors and the reviewers. Any product that may be evaluated in this article, or claim that may be made by its manufacturer, is not guaranteed or endorsed by the publisher.
